# Impact of sublethal zinc exposure on ectomycorrhizal *Laccaria bicolor* x poplar symbiosis

**DOI:** 10.3389/fpls.2025.1656580

**Published:** 2025-09-01

**Authors:** Maarten Ottaway, Janne Swinnen, Katoo Verhaevert, Joske Ruytinx

**Affiliations:** Research Group of Plant Genetics, Vrije Universiteit Brussel, Brussel, Belgium

**Keywords:** *Laccaria bicolor*, poplar, ectomycorrhiza, zinc toxicity, zinc transport, anti-oxidative response

## Abstract

Soil Zn pollution is a widespread problem that is impacting on plant growth and production. Several tree species can rely on fungal ectomycorrhizal symbionts to mitigate toxicity effects to some extent. Here, we explored the impact of Zn pollution on *L. bicolor* and its ectomycorrhizal symbiosis with *Populus tremula x alba*. Next to growth and morphological parameters in sublethal Zn exposure, we investigated responses of symbiosis marker genes, reactive oxygen species scavenging enzymes and Zn transporters in presence and absence of a host plant. Our results indicate that the ECM symbiosis development is maintained in excess Zn conditions despite a reduction in fungal and plant growth. Symbiosis marker gene expression showed sensitivity to Zn excess, even when the fungus was cultured in absence of a host. Zn-induced transcriptional responses of ROS scavenging enzymes and Zn transporters were mainly restricted to mycelia in presence of a host and less prevalent without host. Establishment of new homeostatic equilibria, in particular in presence of a host, seem essential to maintain symbiosis, protect the host and adapt physiologically to Zn pollution. This research furthers our understanding of how resilient plant-fungal symbiotic interactions are, and the interplay between both partners in changing environmental conditions.

## Introduction

Zinc (Zn) is an element, naturally present in soils as a micronutrient. However, presence of high Zn levels in the environment could cause phytotoxicity, resulting in reduced growth and yield (Kaur and Garg, 2021). Worldwide a large number of sites have been identified as surpassing the acceptable threshold value and are considered Zn polluted (Kusuma Wardhani et al., 2022; Han et al., 2023; Van Eynde et al., 2023). Especially industrialized and densely populated areas are rich in Zn polluted sites. Many of these sites are restricted in surface and limited to an area of 10 km surrounding historical mines, smelters or metallurgic industry (Van Eynde et al., 2023). Also urban areas and road sites can be locally enriched in Zn due to corrosion of galvanized materials or particles of car break and tire wear (Blok, 2005). Agricultural soils might show high Zn concentrations due to use of phosphate fertilizers or sewage and grassland soils grazed by cattle due to Zn additives in feed (Van et al., 2024). Regardless of the contamination source, these sites are no longer suited for economically viable plant production and considered wasteland.

The growing population is pressuring optimized land-use and demanding remediation of polluted soils or alternative valorization of this type of wasteland. Repurposing land as recreational area or remediate with energy or wood industry crops could offer a solution (Van et al., 2024). Deep rooting plants such as trees are preferred vegetation since they could prevent leaching and eventually if desired extract part of the Zn (Tan et al., 2023). In particular, fast-growing trees with a certain degree of metal tolerance such as poplars are interesting for bioremediation purpose (Pilipovic et al., 2019; Tozser et al., 2023). Other trees, having a more dense canopy and contributing to alternative ecosystem services (e.g. carbon storage, water retention, temperature cooling, timber) can be more suited for recreational areas such as parcs and forests in urban areas (Sjöman et al., 2018). However, trees such as oak (*Quercus* sp.) and pine (*Pinus* sp.) are sensitive to high soil Zn concentrations (Jordan, 1975; Ivanov et al., 2024). But also in *Populus trichocarpa* it was found that Zn pollution caused a reduction of both leaf and root biomass (De Oliveira and Tibbett, 2018).

The majority of tree species with temperate and boreal distribution engages in ectomycorrhizal (ECM) symbiosis (Tedersoo and Brundrett, 2017). ECM symbiosis is a mutual beneficial partnership between plant roots and certain soil borne fungi. The symbiotic structure that is formed, is composed of a hyphal mantle surrounding tree root tips, and a Hartig net consisting of fungal hyphae penetrating apoplastic space in between epidermal and cortical cells. Extraradical hyphae extend from the root tip into the soil (Genre et al., 2020). A delicate molecular crosstalk between plant and fungal partner is required to accommodate the fungus and maintain the symbiosis (Martin et al., 2016). Most of the molecular work, deciphering this crosstalk was done in the *Laccaria bicolor* – *Populus tremula x alba* ectomycorrhizal system. In this model, the crosstalk includes the production of a series of fungal mycorrhizal induced small secreted proteins (MiSSPs) to regulate plant transcriptional responses and silence immune system next to enzymes and proteins to modify cell walls (Plett et al., 2014; Daguerre et al., 2020; Kang et al., 2020; Zhang et al., 2021a; Marqués-Galvez et al., 2024). At the fungal side, regulation of reactive oxygen species (ROS) levels was shown to be required for proper development of the Hartig net (Shi et al., 2024). Interestingly, environmental stressors such as Zn pollution are well known modifiers of ROS metabolism in plant and fungus (Stanton et al., 2022). While ROS cause cellular damage, they, and specifically H_2_O_2_, also have a role as signaling molecule that helps trigger the plant abiotic stress response. This allows the plant to increase its production of ROS scavenging enzymes, and also adapt to limit the uptake of metal pollutants (Lin and Aarts, 2012). The interaction of both, Zn pollution and presence of a host plant on ROS metabolism and by extension ECM establishment was not explored so far.

While the fungus provides a larger surface area from which nutrients and water can be scavenged, it receives plant-derived sugars in return (Genre et al., 2020). Also, ECM fungi have been shown to provide additional benefits and protect their host plant from metal, including Zn, toxicity. They limit the transfer of excess Zn towards the host plant. In particular the fungal mantle surrounding root tips has a crucial role in blocking excess metals (Zhang et al., 2021b; Quan et al., 2023). In case of Cd and Cu, a large fraction of the accumulated metals will be sequestered by the fungal cell wall (Quan et al., 2023). However, cell wall binding capacity is confined, and it was shown that exposure to high external Zn results in build-up of intracellular Zn concentrations over time (Ruytinx et al., 2013). This internal Zn needs to be managed to prevent cellular damage. Effective management of intracellular Zn requires activation of transmembrane transport for export or storage into organelles and/or reduction of transport from the environment or organelles towards the cytosol (Ruytinx et al., 2020). Transporters of the Zrt-Irt like protein (ZIP) and Cation Diffusion Facilitator (CDF) family were shown to be predominant in eukaryotic transmembrane Zn trafficking (Bui and Inaba, 2024). Few CDF and ZIP transporters of ECM fungal species were functionally characterized and display functions in cellular Zn homeostasis (Blaudez and Chalot, 2011; Coninx et al., 2017; Ruytinx et al., 2017; Coninx et al., 2019; Ho-Plagaro et al., 2024). Their role at the plant-fungal interface and eventual plant protection was not investigated.

In the present study we explore the impact of excess environmental Zn on establishment of ECM symbiosis using the *Laccaria* – poplar model system. We investigated both the effect of excess Zn on morphological characteristics and known molecular determinants. Furthermore, we identified Zn transporters specifically regulated by the presence of a host plant and showing differential response upon Zn exposure as candidates with function in host protection. All together our data provide a solid base for future functional genetic studies in order to come to a better understanding of how ECM symbiotic relationship responds and functions in a Zn polluted environment.

## Materials and methods

### Fungal material, culture conditions and dose-response experiment

*Laccaria bicolor* S238N (Maire) P.D. Orton was used in all experiments. Fungal cultures were grown at 23°C in the dark and maintained by regular subculturing on modified Pachlewski P5. P5 medium is composed of 0.5 g l^-1^ di-NH_4_-tartrate, 1 g l^-1^ KH_2_PO_4_, 0.5 g l^-1^ MgSO_4_.7H_2_O, 5 g l^-1^ maltose, 20 g l^-1^ glucose, 0.1 mg l^-1^ thiamine-HCl, 5 mg l^-1^ MnSO_4_.4H_2_O, 8.5 mg l^-1^ H_3_BO_3_, 0.3 mg l^-1^ (NH_4_)_6_Mo_7_O_24_.4H_2_O, 6 mg l^-1^ FeCl_3_, 0.6 mg l^-1^ CuSO_4_.5H_2_O, 2.7 mg l^-1^ ZnSO_4_.7H_2_O and 20 g l^-1^ agar at pH 5.5 (Kemppainen and Pardo, 2011). Zn tolerance was evaluated via dose-response experiments. Inocula (0.5 cm² plugs) were transferred to cellophane-covered petri dishes (9cm diameter) of P5 medium or P5 medium enriched with ZnSO_4_·7H_2_O. Final Zn concentrations in the medium were as follows: 0.008 (control), 0.05, 0.1, 0.2, 0.4, 0.6, 0.8, 1, 2, 3, 4, 8, 12, 16, 20 and 30 mM Zn. Metal exposures were performed with five replicates per concentration. After three weeks, mycelia were harvested, weighed and lyophilized. Dry weights were determined and a non-linear regression with four parameter log-logistic model (Ritz et al., 2015) was fitted to the data using “R” version 4.4.1 (R Core Team, 2024). Maximal effective concentration (EC50) values were deduced and represent the concentration that inhibits growth by 50% compared to the control condition.

### Fungal – plant co-cultures

*Populus tremula* x *alba* 717-1B4 plants were maintained through micropropagation on Murashige and Skoog medium (MS) supplemented with 10 µM IBA (Murashige and Skoog, 1962) and subsequently used to set-up an *in vitro* co-culturing experiment between *L. bicolor* S238N and *P. tremula* x *alba* 717-1B4. This was established as previously described (Felten et al., 2009). Both mycelium grown in the presence/absence of a host plant were grown on sugar-reduced Pachlewski P20 medium containing 0.1% MES, where incubation occurred at 23°C with a photoperiod of 16h for 14 days. This P20 medium contained either a control condition (7.89 µM ZnSO_4_.7H_2_O) or a sublethal Zn concentration (1.5 mM ZnSO_4_.7H_2_O).

After two weeks of growth, scans of complete co-culture systems were made using a CanoScan 9000F Mark II scanner (Canon) and both plant and fungal material were harvested. Roots were fixated overnight at 4°C in 4% paraformaldehyde. These were subsequently washed and stored in phosphate buffered saline (PBS) awaiting microscopic analysis. Lastly, five biological replicates of mycelium cultured with (mixed plant–fungal material including root, mycorrhizal root tip and mycelium within 5 mm distance of a root) or without a host plant were collected for further RNA extractions (100 mg) and enzyme activity assays (100 mg), flash frozen in liquid nitrogen and stored at -80°C.

### Analysis of root growth and mycorrhizal morphology

Total root length per plant was determined based on scans using RhizoVision Explorer (Seethepalli et al., 2021). The amount of root tips per plant were manually counted and mycorrhizal state was evaluated. The number of root tips per length unit (total root tips/total root length) and percentage of mycorrhization (mycorrhized root tips/total root tips) was calculated. The Mantle thickness and Hartig Net depth of mycorrhized root tips 14 days postinoculation (dpi) was determined. Fixed roots were embedded in 4% agarose, and 25 µm thick cross-sections were made using a VT1000S vibratome (Leica; 0.225 mm/s – 40 Hz). Sections located 275 µm from the start of the root tip were stained with 10 µg/ml Wheat Germ Agglutinin Alexa fluor 488 (WGA-488; Invitrogen W11261) for 1h, after which counterstaining with 15 µM Propidium Iodide (PI; Invitrogen P3566) for 30 min was performed. Stained sections were visualized using an Eclipse Ti2 inverted fluorescence microscope (Nikon). At four different points per sample, the Mantle thickness and Hartig Net depth were determined using the NIS-elements software (Nikon). A minimum of five root tips (biological replicates) were analyzed. Differences among treatments were assessed by a t test at 0.05 significance level, performed in R Studio (Version 2024.4.2.764).

### RNA extraction, cDNA synthesis and quantitative real-time PCR

RNA was extracted using the RNeasy Plant Mini kit (Qiagen). First, 100 mg of material was ground in liquid nitrogen using a pestle and mortar. Next, the subsequent steps of the manufacturers protocol were followed. Additionally, 2% PEG8000 was included in the RLC buffer, and the DNase treatment was incorporated into the protocol. The eluted RNA was flash frozen in liquid nitrogen and kept at -80°C.

The integrity and purity were verified using the Bioanalyzer 2100 (Agilent Technologies) and the NanoDrop One Spectrophotometer (Thermo Fisher Scientific), respectively. Of the approved RNA, 250 ng was converted to cDNA using the High-Capacity cDNA reverse transcription kit and the accompanying protocol (Applied Biosystems).

Expression of marker genes for ectomycorrhizal symbiosis in both *L. bicolor* and *P. tremula x alba*, specifically *TPS16* (Terpene synthase; ID: PtXaTreH.01G253600.1), *TPS21* (Terpene synthase ID: PtXaTreH.19G012400), *GH28a* (endopolygalacturonase, ID: 613299), *MiSSP7* (mycorrhizal induced small secreted protein, ID: 298595) and *MiSSP17* (mycorrhizal induced small secreted protein, ID: 332226) was determined. Besides, gene expression of reactive oxygen species decomposing enzymes, *CAT* (catalase, ID: 123238), *Mn/Fe SOD1* (super oxide dismutase, ID: 635077), *Mn/Fe SOD2* (ID: 192586), *Mn/Fe SOD3* (ID: 291347), *Mn/Fe SOD4* (ID: 295682) and *Mn/Fe SOD5* (ID: 312019) was measured. The expression of predicted Zn transporters in *L. bicolor* was also measured. The *L. bicolor* genome counts four predicted Zn transporters of the CDF family *CDF-A* (ID: 305317), *CDF-B* (ID: 307944), *CDF-C* (ID: 625478) and *CDF-*D (ID: 191080), and five predicted Zn transporters of the ZIP family *ZIP-A* (ID: 180140), *ZIP-B* (ID: 305445), *ZIP-C* (ID: 309863), *ZIP-D* (ID: 189929) and *ZIP-E* (ID: 309134). Gene expression was assessed on five biological replicates four each experimental condition. A no-template control was included in the experimental design. The mentioned real-time reverse transcription PCR (qRT-PCR) reactions were executed in 20 µl reactions containing 2x GoTaq qPCR Master Mix (Promega), 400 nM FW and RV primers and 2 µl of the produced cDNA. Nuclease free H_2_O was added for a total volume of 20 µl. The mixture was then transferred into 96- well plates and the following cycling program was run on the MyiQ™2 (Bio-Rad): 1 cycle of 95°C for 3 min and 40 cycles of 95°C for 10 seconds and 60°C for 30 seconds. All primers used in this study can be found in **Supplementary Table S1**.

Multiple reference genes originating from Pellegrin et al., 2019 were tested using the above-mentioned protocol. Thereafter they were analyzed using the Genorm feature of the qBase+ software to assess their stability in the used growth conditions. The recommended genes (Mycocosm protein ID: 313997 and 446085) were used to calculate a normalization factor (NF) and determine expression levels of the gene of interest following the formula: 2 ^(Ct-Ctmin)/^NF (Vandesompele et al., 2002). These values were then log2 transformed, the Shapiro test was performed, and normality of all data was assessed. To assess differences among treatment groups, this was followed by a two-way ANOVA with a TukeyHSD posthoc test, performed in R studio.

### ROS-scavenging enzyme activity assays

The catalase (CAT) and superoxide dismutase (SOD) enzyme activity was determined. The samples (100 mg) were crushed in liquid nitrogen using a pestle and mortar. To the sample powder, 750 µl of extraction buffer (0.1 M TRIS, 1 mM Na_2_-EDTA, 1 mM DTT, pH 7.8), supplemented with 20 mg PVP-40, was added. This was followed by a 10 min centrifugation step at 13000 RPM and 4°C. Assays consisted of five biological replicates per condition, and three technical replicates per biological replicate.

To determine the CAT enzyme capacity within the samples, the decrease in absorbance over time at 240 nm (H_2_O_2_) was measured. Using the Lambert-Beer law, the decrease in absorbance at 240 nm over time and the amount of sample used, the CAT enzyme capacity (U/g) was determined (Aebi, 1974). The SOD enzyme capacity was calculated by measuring the increase in absorbance at 550 nm (reduced Cyt C) over time. It is defined that one unit of SOD inhibits cytochrome C reduction by 50%. By measuring the inhibition of cytochrome C reduction by the presence of sample SOD, the SOD enzyme capacity (U/g) can be calculated using the Lambert-Beer law (McCord and Fridovich, 1969). Using the Shapiro test, normality of both the calculated CAT and SOD enzyme capacity data was checked. These data were then subjected to a two-way ANOVA with a TukeyHSD posthoc test, which was performed in R studio.

## Results

### Effect of Zn exposure on fungal growth, mycorrhiza development and morphology

**Figure 1:**
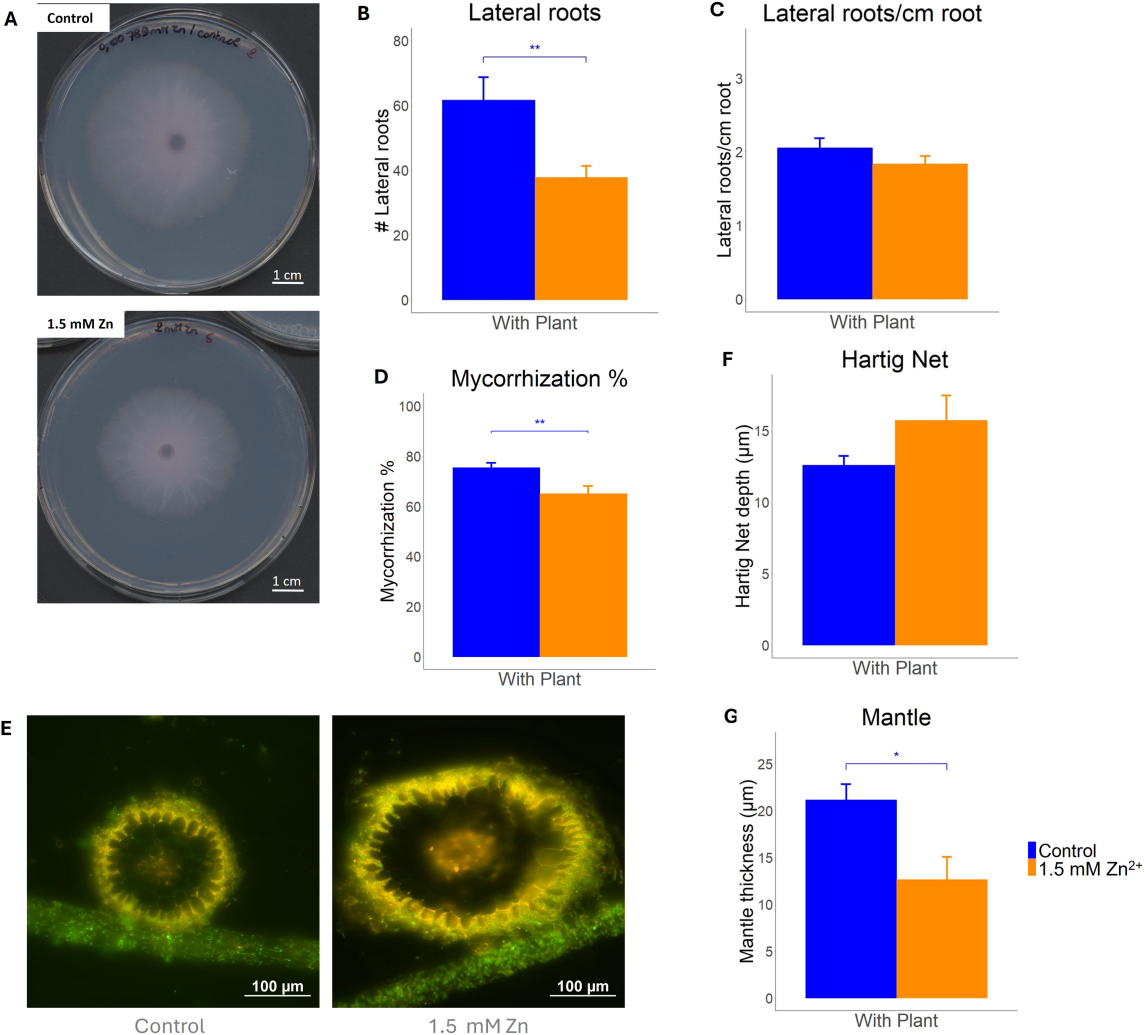
Ectomycorrhiza formed between *L. bicolor* S238N and *P. tremula x alba* 717-1B4 and grown under control (blue) and 1.5 mM Zn (orange) conditions for 14 days. **(A)** *L. bicolor* S238N mycelium grown on P5 medium supplemented with 0.008 mM Zn/control **(Top)** and 2 mM Zn (Bottom). **(B)** Average amount of lateral roots per plant grown under control or 1.5 mM Zn conditions (n ≥ 25). **(C)** Average ratio of lateral roots/cm root measured in roots (14 dpi) grown under control or 1.5 mM Zn (n ≥ 24). **(D)** Average degree of mycorrhization of Poplar roots grown under control or 1.5 mM Zn (n ≥ 24). **(E)** Fluorescent microscopy image of mycorrhized root cross-sections from control (Left) or 1.5 mM Zn (Right) samples stained with WGA-488 and counterstained with PI. Scale bar = 100 µm. **(F)** Hartig Net depth and **(G)** Hyphal Mantle thickness measured in WGA-488 and PI-stained mycorrhiza samples (n ≥ 8). Four measurements were made per sample. Statistical analyses were performed in R Studio using a Student’s t-test with *p<0.05 and **p<0.01. Means of all replicates + standard error (SE) are shown.

To determine the impact of sublethal Zn on both the fungus and plant-fungus symbiosis, fungal growth and the ECM symbiosis morphology were examined. First, increasing Zn concentration in the culture media resulted in growth inhibition of *L. bicolor* (**Figures 1A** and S1). An EC50 of 4.8 mM was determined, at a concentration of 12 mM growth was nearly absent. Therefore, a concentration of 1.5 mM Zn, not resulting in any major growth defects, was considered sublethal for the fungus and used in co-culture experiments (**Supplementary Figure S1**). In co-culture, *P. tremula x alba* root growth was inhibited by 1.5 mM Zn. Plants grown in presence of Zn showed significantly less root tips compared to plants grown in control conditions (**Figure 1B**). However, the number of root tips per length unit of root remained stable regardless of the Zn treatment (**Figure 1C**). The percentage of mycorrhized root tips was significantly lower upon Zn treatment (**Figure 1D**). Root tip sections confirmed normal morphology for mycorrhizas, including a mantle and Hartig net, upon Zn treatment (**Figure 1E**). The average Hartig Net depth was measured to be 12.6 ± 1.0 µm in control conditions, whereas in samples exposed to 1.5 mM Zn it was determined to be 15.7 ± 1.4 µm. This change in average depth was found not to be significant (**Figure 1F**). The average hyphal mantle thickness of control samples was measured to be 21.1 ± 2.7 µm. For samples exposed to Zn excess, the average thickness was 12.7 ± 2.5 µm, which was significantly lower compared to the average thickness of control samples (**Figure 1G**).

### Effect of Zn exposure on symbiosis marker gene expression in presence and absence of a host plant

**Figure 2:**
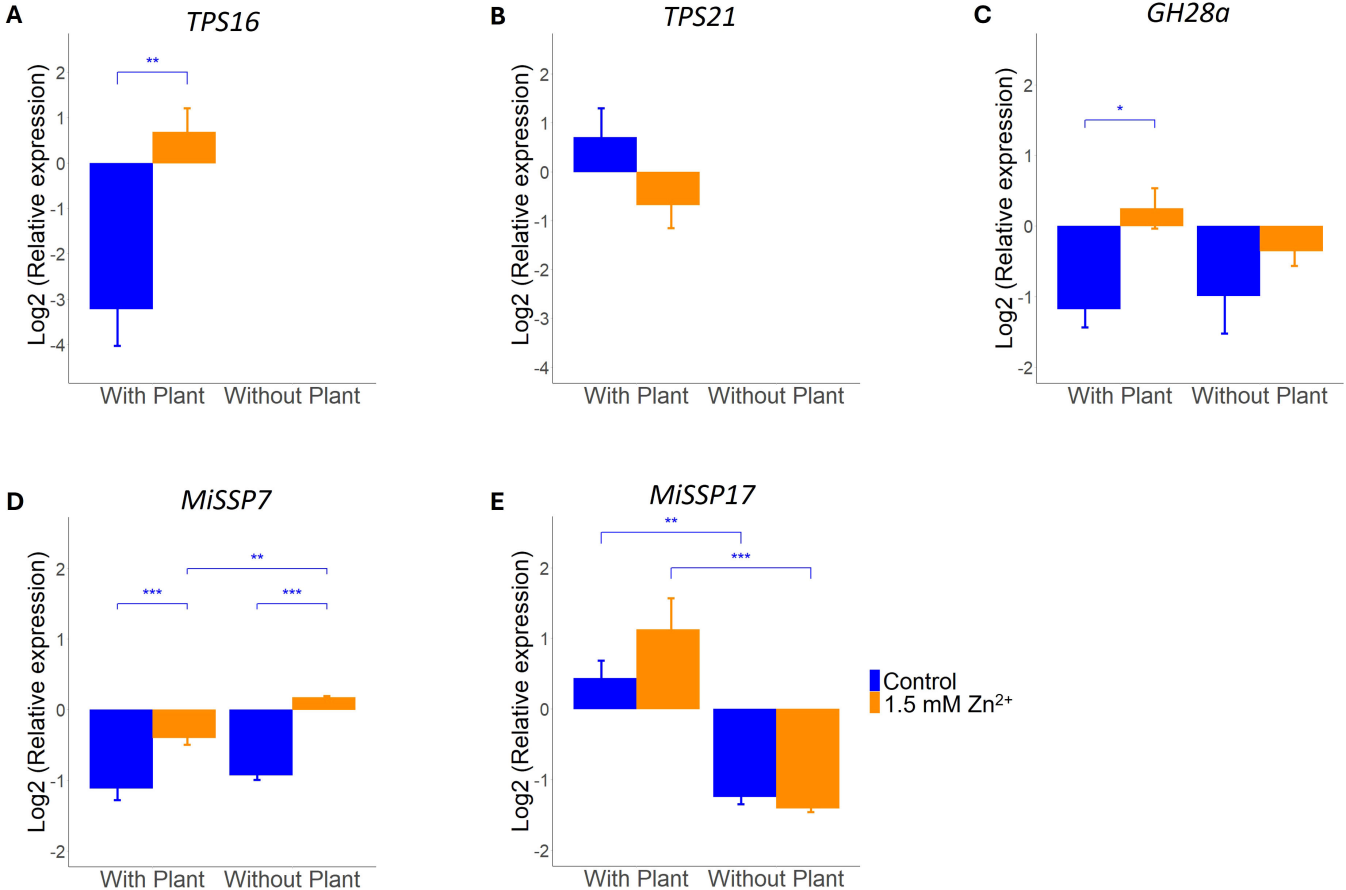
qRT-PCR analysis of symbiosis marker genes in *L. bicolor* S238N and *P. tremula x alba* 717-1B4 (14 dpi) under control (blue) or 1.5 mM Zn (orange). Relative expression of **(A)** poplar *TPS16* (n = 4-5) and **(B)** *TPS21* (n = 5), as well as **(C)** fungal *GH28a* (n = 5), **(D)** *MiSSP7* (n = 5) and **(E)** *MiSSP17* (n = 5). Statistical analyses were performed in R Studio using a two-way ANOVA with TukeyHSD posthoc test. *p<0.05, **p<0.01 and ***p<0.001. Means of all replicates per target gene + SE are shown.

While only a slight change in ECM morphology was found, Zn exposure could have a more profound effect on the expression of symbiosis marker genes. Expression of two poplar terpene synthase (TPS) genes was measured. As *TPS* genes are exclusively expressed in poplars in this symbiosis, expression was only found in samples cultured in the presence of a host plant. Only *TPS16* expression was affected by exposure to excess Zn; *TPS21* expression remained stable across Zn treatments (**Figures 2A, B**). Besides several *L. bicolor* genes known to be involved in establishment of ectomycorrhizas were measured. ANOVA analysis showed that exposure to excess Zn is impacting on *MiSSP7* and *GH28* expression. No interaction effects (Zn x host) were detected (**Supplementary Table S2**).

Expression of *GH28a*, a CAZyme expressed by *L. bicolor* throughout symbiosis development, was found to be significantly impacted by Zn exposure. This increase was only significant in the presence of a plant (**Figure 2C**) despite visibility of a similar trend in absence of the host. For *MiSSP7* the increase in expression upon exposure to excess Zn was significant in both presence/absence of a host plant (**Figure 2D**). *MiSSP17* expression, however, was not affected by excess Zn and only induced by the presence of a host plant (**Figure 2E**).

### Effect of Zn exposure on ROS producing and decomposing enzymes in presence and absence of a host plant

**Figure 3:**
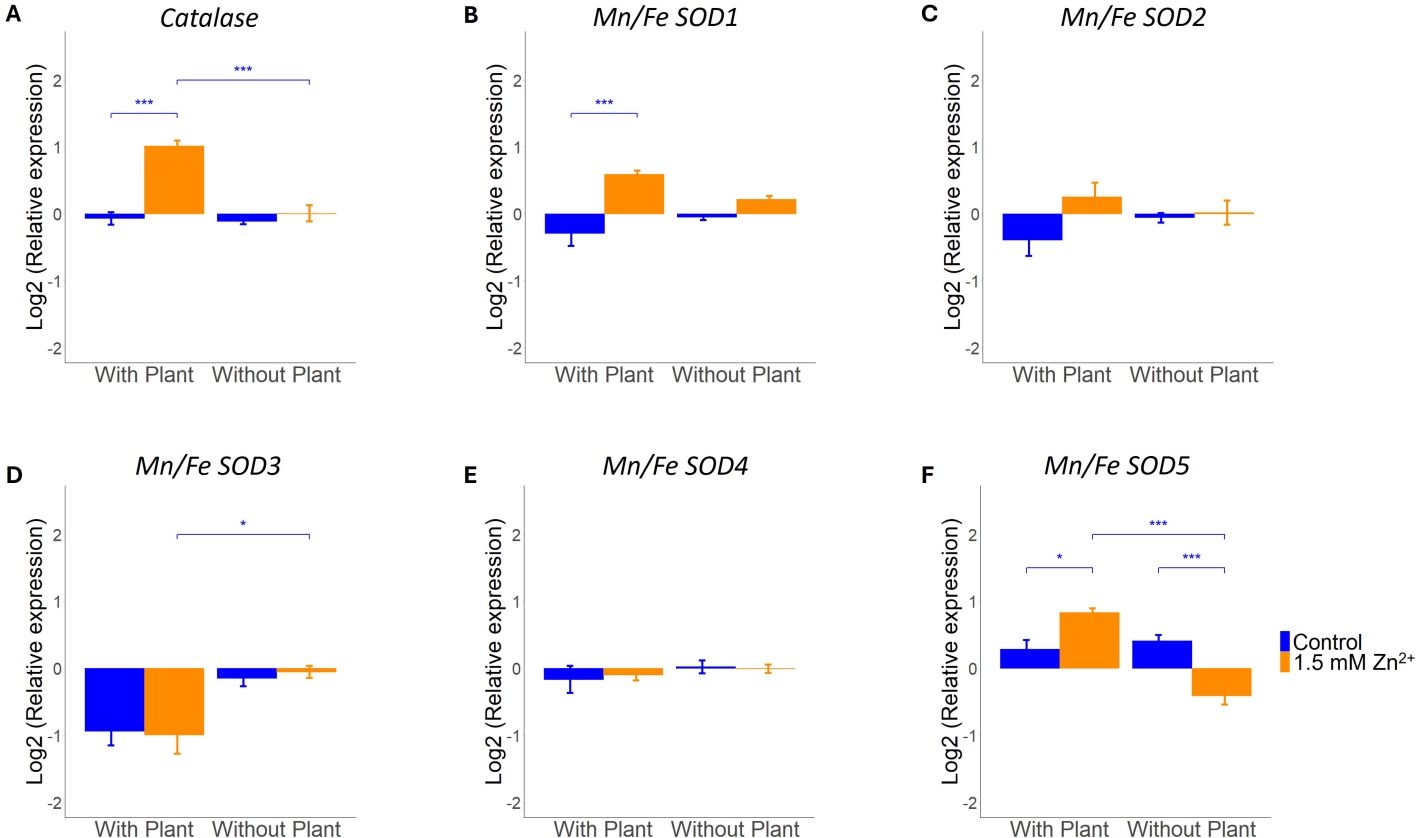
qRT-PCR analysis of *L. bicolor* S238N ROS scavenging genes (14 dpi) under control (blue) or 1.5 mM Zn (orange). Relative expression of **(A)** *catalase* (n = 5), **(B)** *Mn/Fe SOD1* (n = 5), **(C)** *Mn/Fe SOD2* (n = 5), **(D)** *Mn/Fe SOD3* (n = 5), **(E)** *Mn/Fe SOD4* (n = 5) and **(F)** *Mn/Fe SOD5* (n = 5) in presence and absence of a host plant. Statistical analyses were performed in R Studio using a two-way ANOVA with TukeyHSD posthoc test. *p<0.05, **p<0.01, ***p<0.001. Means of all replicates per target gene + SE are shown.

**Figure 4:**
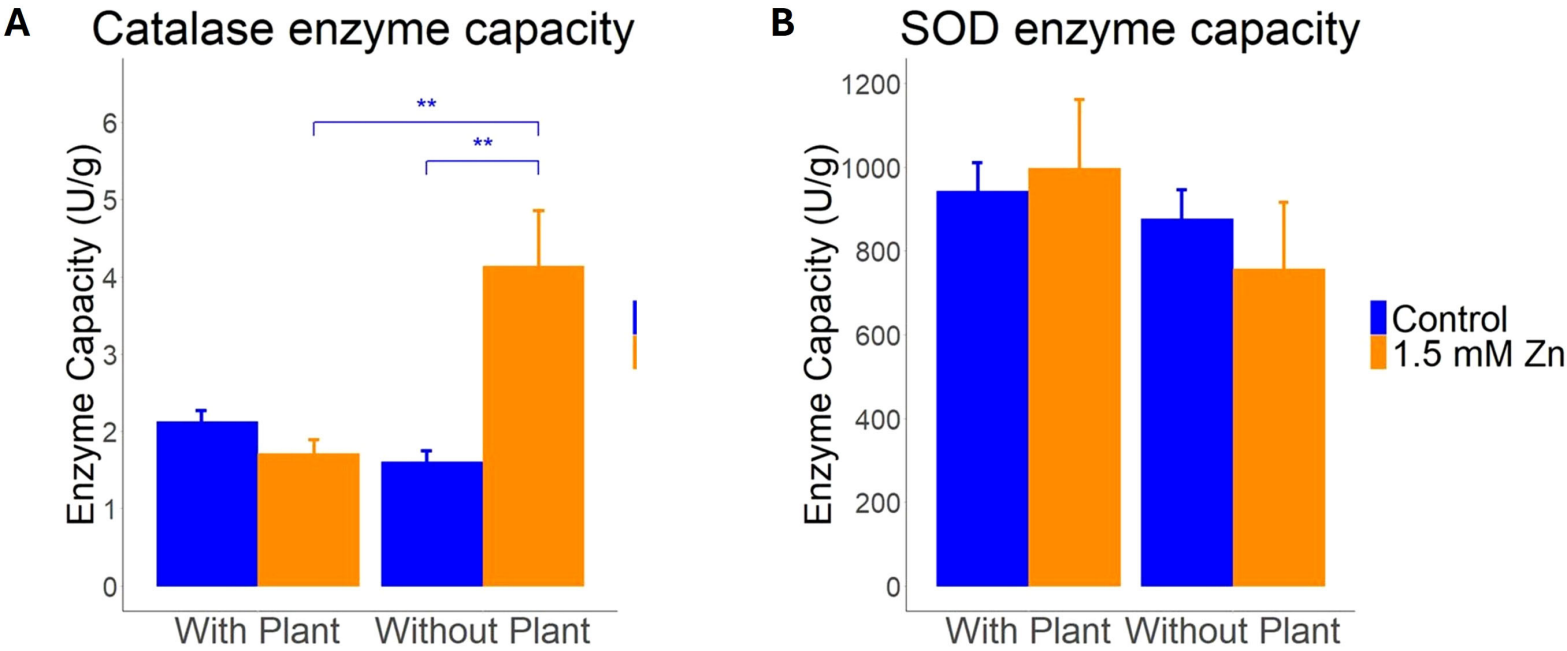
Enzyme capacity assays for **(A)** Catalase (n = 5) and **(B)** Superoxide dismutase (n = 5). Whole cell extracts from 100 mg of mycelium grown in presence or absence of a host plant, cultured for 14 days under control or 1.5 mM Zn conditions, were used. Statistical analyses were performed in R Studio using a two-way ANOVA with TukeyHSD posthoc test. ** = p<0.01. Means of all replicates for CAT or SOD + SE are shown.

As exposure to excess Zn and symbiosis development may result in oxidative stress, regulation of ROS scavenging genes by both factors and their interaction was investigated (Baptista et al., 2007; Branco et al., 2022). Gene expression of predicted *L. bicolor* catalases (*CAT*) and superoxide dismutases (*SOD*) was analyzed next to overall capacity for both enzyme groups in presence and absence of a host plant for control and sublethal Zn treatments. The *L. bicolor* genome counts one predicted CAT and five SODs. Two-way ANOVA indicated CAT gene expression and enzyme capacity to be impacted by Zn treatment, presence of a host plant and interaction of both factor (Zn x host) (**Figures 3A**, **4**; **Supplementary Table S2**). Specifically, *CAT* gene expression was found to be significantly upregulated after Zn exposure, but only in tissue cultured in the presence of a host plant (**Figure 3A**). The total plant-fungal CAT enzyme capacity was also increased in mycelium exposed to excess Zn, but only in tissue cultured in absence of a host plant (**Figure 4A**).

There are five predicted *Mn/Fe SOD* genes in the *L. bicolor* genome, of which two (*Mn/Fe SOD2* and *Mn/Fe SOD4)* were not impacted by the tested conditions (**Figures 3C, E**; **Supplementary Table S2**). *Mn/Fe SOD1* expression was impacted by Zn treatment and showed an interaction effect of Zn treatment and presence of a host plant (Zn x host; **Supplementary Table S2**). It was found to be increased after Zn exposure in co-culture with a host plant (**Figure 3B**). *Mn/Fe SOD3* and *Mn/Fe SOD5* expression was impacted by the presence of a host plant (**Supplementary Table S2**). *Mn/Fe SOD3* showed a decreased expression in co-culture conditions, regardless of Zn treatment (**Figure 3D**). Besides, an interaction effect of both evaluated factors (Zn x host) was detected for *Mn/Fe SOD5* (**Supplementary Table S2**)*. Mn/Fe SOD5* expression was affected differently by Zn treatment depending on the presence/absence of a host plant (**Figure 3F**). Analysis of the total SOD enzymatic capacity did not reveal a significant change, regardless of Zn treatment or presence/absence of a host plant (**Supplementary Table S2**, **Figure 4B**).

### Effect of Zn exposure on Zn transporter expression in presence and absence of a host plant

**Figure 5:**
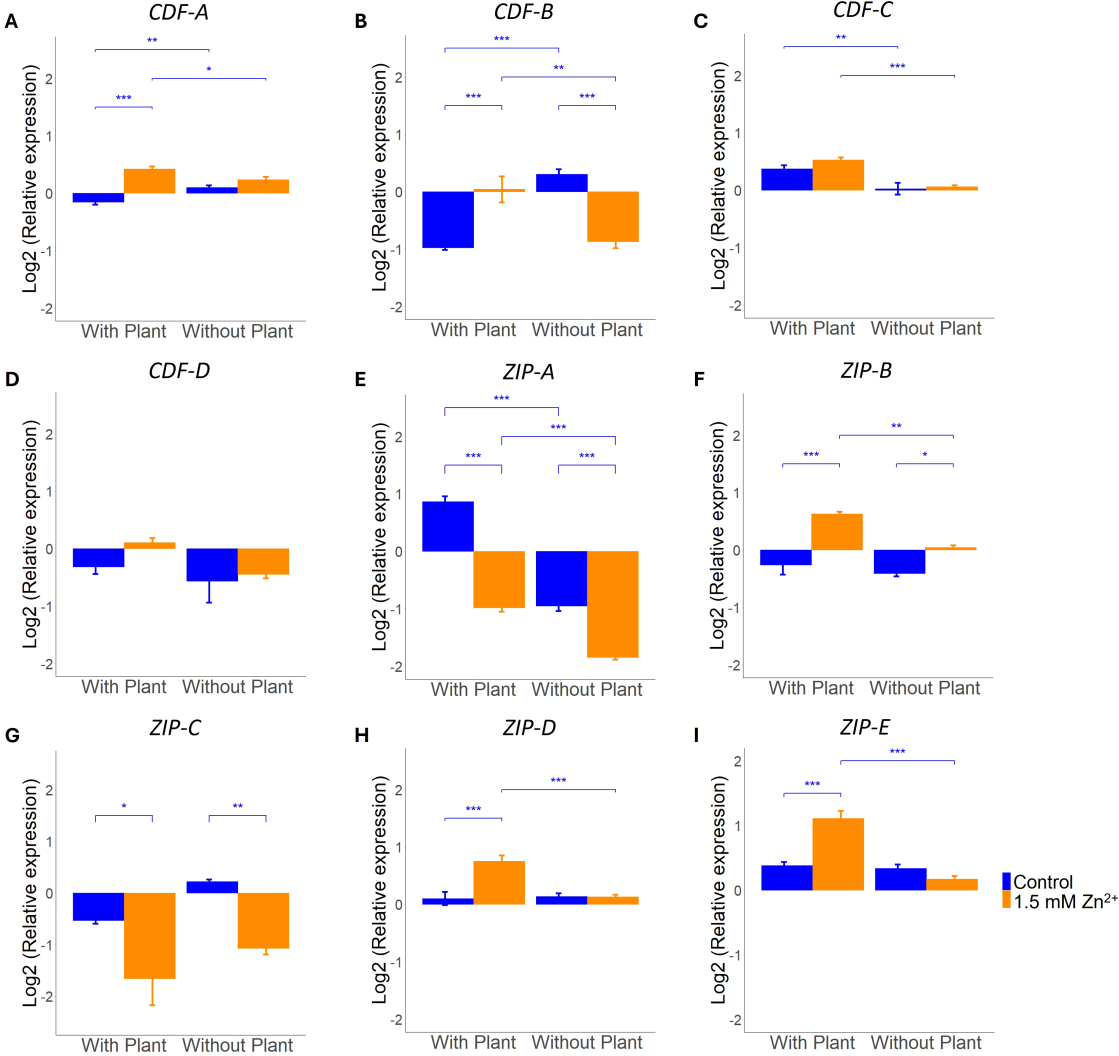
qRT-PCR analysis of *L. bicolor* S238N *CDF* and *ZIP* transporter genes after 14 days of incubation under control (blue) or 1.5 mM Zn (orange). Relative expression of **(A)** *CDF-A* (n = 5), **(B)** *CDF-B* (n = 5), **(C)** *CDF-C* (n = 5), **(D)** *CDF-D* (n = 5), **(E)** *ZIP-A* (n = 5), **(F)** *ZIP-B* (n = 5), **(G)** *ZIP-C* (n = 5), **(H)** *ZIP-D* (n = 5) and **(I)** *ZIP-E* (n = 5) in mycelium grown in presence or absence of a host plant. Statistical analyses were performed in R Studio using a two-way ANOVA with TukeyHSD posthoc test. *p<0.05, **p<0.01, ***p<0.001. Means of all replicates per target gene + SE are shown.

Another strategy that can be employed to maintain an optimal concentration of intracellular Zn and deal with sublethal Zn exposure, is through modulating the expression of Zn transporters (Ruytinx et al., 2020). We explored the role of different *L. bicolor* CDF and ZIP family transporters, annotated as Zn transporters, in maintenance of cellular Zn homeostasis in presence and absence of a host plant. Four *CDF* transporter genes, these being *CDF-A* through to *CDF-D*, and five *ZIP* genes, being *ZIP-A* to *ZIP-E*, can be found in the *L. bicolor* genome. Of the four *CDF* genes, *CDF-A* is the only one impacted by the factor Zn treatment according to the two-way ANOVA analysis. Besides, this gene is showing an interaction effect (Zn x host) as also detected in *CDF-B* (**Supplementary Table S2**). Tukey *post-hoc* test indicated *CDF-A* to be higher expressed after Zn exposure under co-culture conditions (**Figure 5A**). The same response was detected for *CDF-B* expression in presence of a host plant whereas an opposite response is detected in absence of a host (**Figure 5B**). Both *CDF-C* and *CDF-D* expression were impacted by presence of a host only as indicated by two-way ANOVA (**Supplementary Table S2**). Nevertheless, Tukey *post hoc* did not detect any significant differences between treatment groups for *CDF-D* (**Figure 5D**). Expression of *CDF-C* was increased in the presence of a host plant but remained unaffected by exposure to excess Zn (**Figure 5C**).

Expression analysis of all five predicted *ZIP* genes showed an impact by the factor Zn treatment and presence of a host plant. An interaction effect of both factors was detected for all except for ZIP-C (**Supplementary Table S2**). Specifically, *ZIP-B*, *ZIP-D* and *ZIP-E* all showed increased expression in mycelium grown in presence of a host plant after Zn exposure. Of these three genes, only *ZIP-B* showed a slight increase in absence of a host plant after Zn exposure (**Figures 5F, H, I**). Expression analysis of *ZIP-A*, rather, revealed a decrease in expression after Zn exposure (**Figure 5E**). *ZIP-C* expression also showed a downregulatory response after Zn exposure, but a host plant being present or not did not cause any further change in the expression of this gene (**Figure 5G**).

## Discussion

### Excess Zn impacts on fungal growth, mycorrhiza development and morphology

Exposure to toxic levels of metals, including Zn, may lead to cell damage, reduced growth, decreased fitness, and eventually death (Branco et al., 2022). Some species, including fungal, develop strategies to withstand metal rich environments and thus the metal concentration they can tolerate in their growth substrate varies among species and across isolates. For example, Zn tolerance has been shown to be an adaptive trait in ectomycorrhizal Suilloid fungi whereas their relative *Paxillus involutus* shows constitutive Zn tolerance (Colpaert et al., 2004). *L. bicolor* was described previously as a species highly tolerant for Zn pollution (Reddy et al., 2014; Liu et al., 2022). Extreme high concentrations, up to 10 mM Zn in Modified Melin Norkans (MMN) medium, reduced biomass production only for ≈ 30% (Reddy et al., 2014). However, MMN is a nutrient rich medium containing malt extract which might impact on Zn bioavailability and bias comparisons with tolerance indices reported for other species in synthetic less nutrient dense media. In the current study, we estimated the EC50 or concentration of Zn to result in 50% growth reduction in P5 medium to be 4.8 mM for *L. bicolor*. Similar to *P. involutus*, this classifies *L. bicolor* as a species of intermediate Zn tolerance according to the tolerance categories defined by (Colpaert et al., 2004).

As for fungi, elevated Zn concentrations in the environment impact on plant growth (Stanton et al., 2022). Ectomycorrhizal symbiosis may reduce metal toxicity in the plant host by limiting transfer of the toxic metal while maintaining nutrient supply (Zhang et al., 2021b). However, impact of excess Zn on establishment of ectomycorrhizal symbiosis in seedlings was never assessed so far. Early responses of tree roots on the presence of ectomycorrhizal fungi include a stimulation of lateral root formation (Felten et al., 2009). In co-culture conditions, poplar microcuttings showed significantly less lateral roots upon exposure to sublethal Zn concentration for two weeks. However, the number of lateral roots per length unit remained constant regardless of the Zn treatment. This suggests that high Zn concentrations impact root growth in general rather than lateral root formation induced by sesquiterpene and auxine signaling of the fungus and thus initiation of symbiosis. In a next step, roots will become colonized by the fungus (Ruytinx et al., 2021). This colonization process is expected to be impacted by Zn excess since we detected lower percentages of mycorrhization in Zn exposed plants.

Ectomycorrhizal colonization results in a fungal mantle of hyphae surrounding root tips and a Hartig net of mycelium occupying apoplastic space between epidermis and cortex cells (Genre et al., 2020). Interestingly, after 14 days of exposure to 1.5 mM Zn, the mantle significantly decreased in thickness. The Hartig net remained unaffected, showing similar depth regardless of the Zn treatment (**Figure 1B**). Similar results were obtained for *Pinus densiflora* – *L. bicolor* ectomycorrhiza upon exposure to Cd and Cu (Quan et al., 2023). As the hyphal mantle forms the physical barrier between the plant root/Hartig net and the soil, this structure will face most of the excess Zn. This suggests that the decrease in mantle thickness might be caused by a decrease in fungal growth. As only the Mantle was affected, and because the ratio of lateral roots/cm root did not change significantly, these results suggest that excess Zn does not interfere with the signaling needed for ECM development or allows to balance impact on fungal and plant partner to establish a new homeostatic equilibrium. Excess Zn does, still, negatively impact both fungal and plant growth as detected by a reduction of total root tips per plant and mantle thickness.

### Symbiosis marker gene expression is affected by exposure to elevated Zn, even in absence of a host plant

Colonization of roots requires inactivation of plant defense genes. In particular, terpene synthase genes *TPS16* and *TPS21* are downregulated during establishment of the *L. bicolor – P. tremula x alba* symbiosis as a consequence of molecular cross-talk between both partners (Marqués-Galvez et al., 2024). We demonstrated that sublethal concentrations of Zn result in an accumulation of *TPS16* transcripts in *L. bicolor* colonized poplar roots whereas expression of *TPS21* transcripts remained unaffected. This selective upregulation of *TPS16* upon exposure to excess Zn is remarkable. In general, monoterpene mixtures inhibit growth of *L. bicolor* and also individual compounds produced by *TPS21* were reported to result in negative growth effects. However, γ-terpinene the compound forming bulk of the monoterpene mixture produced by *TPS16* in poplar was shown previously to stimulate *L. bicolor* growth (Lackus et al., 2018; Marqués-Galvez et al., 2024).

Establishment of ECM symbiosis also requires reprogramming of fungal transcriptional responses. We studied the effect of sublethal Zn concentrations on three well-established marker genes*: MiSSP17* a mycorrhiza induced small secreted protein of unknown function with ECM specific expression pattern, *MiSSP7* a mycorrhiza induced small secreted protein modifying host immune response and *GH28a* an endopolygalacturonase modifying cell walls and essential for Hartig net development (Plett et al., 2014; Ruytinx et al., 2021; Zhang et al., 2021a). We found all of these genes to be upregulated in response to excess Zn in the presence of a host plant. Interestingly, *MiSSP7* was also induced by excess Zn in absence of a host and *GH28a* showed a similar trend. The upregulation of these genes might indicate the need for increased measures to counteract Zn induced plant defense or detoxification response in order to establish and maintain symbiosis. RNA-seq data of poplar roots exposed to excess Zn showed a downregulation of JAZ6 (Ariani et al., 2015). JAZ6 is a repressor of jasmonic acid (JA) induced plant defense responses and a reduction of its expression will result in activation of the defense response (Chung et al., 2010). Interestingly, this is the opposite as required for ECM establishment in which *MiSSP7* stabilizes JAZ6 and prevents activation of JA-induced defense response (Daguerre et al., 2020; Marqués-Galvez et al., 2024). Also, it has been shown that Zn excess increases pectin production in plant roots, as mechanism to bind excess Zn to the cell wall (Kaur and Garg, 2021). *GH28a* was characterized as an enzyme degrading pectin and polygalacturonic acid which is essential for Hartig net formation (Zhang et al., 2021a).

### Gene expression and enzyme activity of ROS scavenging enzymes show contrasting responses

Compatible fungal-host ectomycorrhizal interactions are strongly associated with pathways involved in the deactivation of ROS and regulation of ROS signaling was shown to be essential for Hartig net development in the *L. bicolor – P. tremula x alba* symbiosis (Baptista et al., 2007; Lofgren et al., 2021). Also exposure to excess Zn is known to induce an anti-oxidative response in mycelium of ectomycorrhizal fungi (Smith et al., 2024). It is not clear how these responses on ROS scavenging enzymes are integrated. We explored the impact of both presence of a host, Zn availability and their interaction on expression and activity of two major classes of ROS scavenging enzymes. Superoxide dismutase (SOD) catalyzes the reduction of superoxide anions into hydrogen peroxide (Kliebenstein et al., 1998). Catalase (CAT) reduces hydrogen peroxide to water (Baker et al., 2023). Both, CAT gene expression and activity were responsive to presence of a host, Zn availability and their interaction. Excess Zn induced expression of CAT in presence of a host only. This might indicate an increase in H_2_O_2_ concentration and thus need for H_2_O_2_ decomposing enzymes particularly in this condition. Surprisingly, this increase in expression was not reflected in an increased activity. Since the fungal sample in the presence of a host also included plant material, the discrepancy between expression and activity might be due to a decrease in plant CAT activity masking an increase in fungal activity. Alternatively, post-transcriptional regulation of the fungal enzyme might have caused the bias. CAT activity is known to be regulated at different levels including protein folding and maturation. Besides, these enzymes are sensitive for different types of posttranslational modifications such as acylation, oxidation and phosphorylation (Baker et al., 2023). Since CAT activity in mycelium in absence of a host significantly increased upon Zn exposure despite any transcriptional response, posttranscriptional regulation of this enzyme is expected to be crucial in determining final cellular ROS levels.

Overall SOD activity was not impacted by Zn availability nor presence of a host plant in our experimental set-up. Expression of several genes annotated as SOD at the other hand was induced by Zn in presence of a host plant (*SOD1, SOD5*) or showed differential expression in presence/absence of a host regardless of the Zn treatment (*SOD3*). Activity measurements represent overall activity including all SODs present in the sample regardless of the gene encoding it (*SOD1-5*) and its nature (plant or fungal). A more detailed monitoring including spatial information or subcellular location will be required to fully understand the functioning of SODs in *L. bicolor* Zn stress and host interaction. Indeed, certain SODs might be secreted and located at the apoplast whereas other show organelle specific location or switch location and function depending on the condition. For example, *Fusarium oxysporum* SOD5 was shown to be an extracellular SOD that is anchored in the membrane and changes location depending on the environmental conditions. Upon limitation of nutrients it switches from a phialide specific location to a more diffuse localization including conidia, septa and hyphae (Wang et al., 2021). Even more impactfully, increases in exogenous or endogenous hydrogen peroxide concentration promote human SOD1 to relocate rapidly from cytosol to nucleus where it changes function towards a transcriptional activator (Tsang et al., 2014). So far, no information regarding subcellular localization nor primary function (reduction of superoxide anion or transcriptional activation) is available for the five *L. bicolor* genes annotated as SOD. Nevertheless, their differential regulation in response to the presence of a host and/or a stress factor despite steady overall activity suggest the importance of subcellular regulation of ROS levels in adaptation towards changing environments.

### CDF and ZIP transporter gene expression is more responsive to excess Zn when in symbiosis with a host plant

Transmembrane transporters are essential for maintenance of cellular homeostasis upon Zn exposure and to manage long distance transport within the mycelial colony or towards the host plant (Ruytinx et al., 2020). *L. bicolor* counts four CDF and five ZIP transporters that are predicted to transport Zn. So far, none of these transporters was functionally characterized and their role in limiting transport of excess Zn towards the host is unknown. We explored transcriptional regulation of these transporters in response to excess Zn, a host and their interaction to pinpoint transporters with a potential function in plant protection. Interestingly, two out of the four CDF family transporters, i.e. *CDF-C* and *CDF-D* were only responsive to the presence of a host and not regulated by external Zn availability (**Supplementary Table S2**). Tukey *post-hoc* confirmed this response for *CDF-C* (**Figure 5C**). Regulation of a Zn transporter of the CDF family by a host plant was detected previously for *HcZnT2* of *Hebeloma cylindrosporum*. This *H. cylindrosporum* ER localized Zn transporter was identified as the transporter gene showing highest transcriptional response, > 100x fold change, upon presence of the host plant *Pinus pinaster* (Ho-Plagaro et al., 2024). The two remaining *L. bicolor CDF transporters* (*CDF-A and CDF-B*), both homologues of *HcZnT2*, displayed an interaction effect in their response towards a host and Zn availability. This interaction effect is visible as a differential regulation by Zn in presence or absence of a host plant, reflected as opposite responses for *CDF-B* (increase with plant, decrease without plant) and an increase in response to Zn in presence of a plant only for *CDF-A*. CDF transporters are known to transport Zn from the cytoplasm towards organelles or extracellular space and in this way contribute at detoxification of excess Zn (Ruytinx et al., 2020). Therefore, an induction of these transporters in response to external Zn availability is expected. However, it is surprising that this response is only detected in presence of a host plant for both *CDF-A* and *CDF-B* and might point to their role in host protection.

In contrast to CDF transporters, we found that all 5 predicted *L. bicolor* ZIP family Zn transporters are regulated by external Zn concentrations (**Supplementary Table S2**). ZIP family transporters transport Zn from the extracellular space or cellular compartments towards the cytosol (Hu and Jiang, 2024). In response to excess environmental Zn a downregulation would be expected in order to control cytoplasmic Zn levels. *L. bicolor ZIP-A* and *ZIP-C* show this expected and typical response both in presence and absence of a host although baseline transcription levels in control conditions are different. Unexpectedly, the other three predicted *L. bicolor* ZIP family Zn transporters show an opposite response. These are induced upon exposure to excess Zn however, in presence of a plant only (*ZIP-D* and *ZIP-E*) or more pronounced in presence then absence of a host (*ZIP-B*). It is puzzling, why the fungus would enhance cytosolic Zn concentration in response to external Zn in presence of a plant. Indeed, two CDF transporters (*CDF-A* and *CDF-B*) are showing the same response and are supposed to have an opposite effect. Alternatively, the increase in expression of ZIP transporters might be not intended to increase cytosolic concentration but rather to prevent build-up of toxic Zn levels in particular organelles. These transporters might be localized on the endomembrane of sensitive organelles and transport excess Zn back to the cytosol in an attempt to protect proper functioning of the organelles. Even so, they might be localized on ER-derived vesicles of the secretory pathway and prevent excess Zn to be secreted at the plant-fungal interface. Functional characterisation of these three *ZIP* genes, including identification of subcellular localization of the encoded proteins, would be required to fully understand their role in maintenance of Zn homeostasis and eventual protective role towards the host plant.

## Conclusion

In conclusion, *L. bicolor* and *P. tremula x alba* are still able to develop ECM symbiosis under Zn stress conditions. However, morphology of the fungal mantle surrounding the root tips is significantly impacted and sublethal Zn concentrations are affecting expression of *L. bicolor* genes essential for ECM establishment even in absence of a host plant. This suggests the need for adjustment of the homeostatic equilibrium in cross-partner signaling in order to allow/maintain symbiosis. Transcription of ROS scavenging enzymes is induced mostly in the presence of a plant only but strongly diverges from the enzyme activity levels recorded, indicating both the importance of posttranscriptional and subcellular regulation of ROS levels. A set of candidate genes encoding putative Zn transporters, potentially involved in managing Zn levels at the plant fungal interface were identified and need further functional characterization to rule out their function in host plant protection.

## Data Availability

The datasets presented in this study can be found in online repositories. The names of the repository/repositories and accession number(s) can be found in the article/supplementary material.
