# Supplementary material for: Impact of sublethal zinc exposure on ectomycorrhizal Laccaria bicolor x poplar symbiosis
Source: Front Plant Sci. 2025 Sep 1;16:1656580. doi: 10.3389/fpls.2025.1656580 (PMC12434098; doi:10.3389/fpls.2025.1656580)
Supplement: Supplementary file 1 [file DataSheet1.docx]

All raw data and metadata are publicly available on Zenodo ([10.5281/zenodo.15181874](https://doi.org/10.5281/zenodo.15181874)). Code used for the statistical analysis is available on Github (https://github.com/mottaway/Laccaria-Poplar-Zn-stress-Code.git).

**Supplementary table S1:** Primers used throughout this study. For poplar genes, the [*Populus tremula x Populus alba* HAP1 v5.1](https://phytozome-next.jgi.doe.gov/info/PtremulaxPopulusalbaHAP1_v5_1) genome on Phytozome was used to obtain their sequence. For *Laccaria bicolor* genes, the Laccaria bicolor V2.0 genome on mycocosm was used.

| Gene target | Protein ID # | Primer sequence (5’ 🡪 3’) |
| --- | --- | --- |
| *TPS16* | PtXaTreH.01G253600.1 | **FW :** CCAACTTGCTCCATTCCCTA  **RV :** TGCTTTGATCACGAGTTTCG |
| *TPS21* | PtXaTreH.19G012400 | **FW :** TTCGACTCCTAAGGCAGCAT  **RV :** ATGCTCTCGCCTTCTACCAA |
| *GH28a*  (Zhang et al., 2021) | 613299 | **FW :** GCAAAGCAGGTTGCGGTAAA  **RV :** CAGTATGTTCGCGTTGCTGG |
| *MiSSP7* | 298595 | **FW :** TCTCGCCTGCACTATCCTCT  **RV :** TTGGGCTCCGGTACTCTTCT |
| *MiSSP17* | 332226 | **FW :** ACCGCCAATGGATGTGCATA  **RV :** CGGTGCAGTGTTGTGTCAAG |
| *Catalase* | 123238 | **FW :** TCAAAACCCAGAGTCGATCC  **RV :** CACAAAGGAACCGTCATCCT |
| *Mn/Fe SOD1* | 635077 | **FW :** ATTCGTTGTTGAGGGAGTGG  **RV :** CGCACATATCAGCGAAAGAA |
| *Mn/Fe SOD2* | 192586 | **FW :** AGGCAATTGAGCGTGACTTT  **RV :** AAGGATCCTGGTTTGCAGTG |
| *Mn/Fe SOD3* | 291347 | **FW :** GGCCACATCAACCACTCTCT  **RV :** GTTATCGAGGCTACCGAACG |
| *Mn/Fe SOD4* | 295682 | **FW :** CCAGGACCCACTTCTCCATA  **RV :** TCAAAGTTGATGACGGACCA |
| *Mn/Fe SOD5* | 312019 | **FW :** CAGTTTGAAGCCAACAAGCA  **RV :** CCAGAACTGCCTCAACCATT |
| *CDF-A* | 305317 | **FW :** GAACTTGTGGGTGCCTTTTTC  **RV :** CTTTGGCGAGGTGATCTTTTC |
| *CDF-B* | 307944 | **FW :** GTTTGGCGTTACTTGCAGATG  **RV :** GGTAGGTTTTCGCGTCTCTTG |
| *CDF-C* | 625478 | **FW :** TGGACGCTTCTTTGCTACTC  **RV :** GCTATGGGGATGTTCGTGAG |
| *CDF-D* | 191080 | **FW :** TCGTAGACACGGGCAAAGTC  **RV :** GCCAGAATCGAGGGAAATCG |
| *ZIP-A* | 180140 | **FW :** CGTCTTCGACTTCGCAAAATAC  **RV :** GGAGAACCAAGCTCGTCTAATG |
| *ZIP-B* | 305445 | **FW :** CCTTCACAGCGTCCTGATTG  **RV :** CCCAGTCCCTCGAATGTTTG |
| *ZIP-C* | 309863 | **FW :** CCTTCCCAGGTCTCTCTAAGC  **RV :** AGATCGAAAAGCGTCATCAAGTAG |
| *ZIP-D* | 189929 | **FW :** AGGAGTGCTCTCGGTTTTC  **RV :** AATAGGCGACTCCTGGTCC |
| *ZIP-E* | 309134 | **FW :** GCCAACCTAGCCAAGACAAC  **RV :** GCGATGTGGGTGCTTTATG |
| *HK gene 1*  (Predicted ribosomal RNA adenine demethylase)  (Pellegrin et al., 2019) | 313997 | **FW :** GAGCAGAGCGGGTACGAATG  **RV :** ACCCGGCCGTACTGGAATAA |
| *HK gene 2*  (Ubiquitin and ubiquitin-like proteins)  (Pellegrin et al., 2019) | 446085 | **FW :** CAAAGCCGCCTACGCCTAAA  **RV :** GGCATAGTGCCTGCATCGAG |

**Supplementary table S2:** ANOVA output for *L. bicolor* x *P. tremula* x *alba* symbiosis marker gene expression, *L. bicolor* antioxidant response gene expression/enzyme activity and *L. bicolor* zinc transporter gene expression.

|  | **Zn x Host** |  | **Zn** |  | **Host** |  |
| --- | --- | --- | --- | --- | --- | --- |
| *GH28a* | F (1, 15) =1.491 | P=0.24093 | F (1, 15) =10.635 | P=0.00526 | F (1, 15) =0.287 | P=0.60007 |
| *MiSSP7* | F (1, 16) =3.528 | P=0.07867 | F (1, 16) =78.518 | P=1.44e-07 | F (1, 16) =13.604 | P=0.00199 |
| *MiSSP17* | F (1, 16) =2.755 | P=0.116 | F (1, 16) =1.030 | P=0.325 | F (1, 16) =66.707 | P=4.23e-07 |
| *CAT*  *Mn/Fe SOD1*  *Mn/Fe SOD2*  *Mn/Fe SOD3*  *Mn/Fe SOD4*  *Mn/Fe SOD5*  CAT capacity  SOD capacity  *CDF-A*  *CDF-B*  *CDF-C*  *CDF-D*  *ZIP-A*  *ZIP-B*  *ZIP-C*  *ZIP-D*  *ZIP-E* | F (1, 16) =28.64  F (1, 16) =9.09  F (1, 15) =2.247  F (1, 15) =0.141  F (1, 16) =0.149  F (1, 16) =39.619  F (1, 16) =14.823  F (1, 16) =0.493  F (1, 16) =24.650  F (1, 16) =64.083  F (1, 15) =1.123  F (1, 14) =0.663  F (1, 16) =39.66  F (1, 16) =5.453  F (1, 15) =0.124  F (1, 16) =15.30  F (1, 16) =35.00 | P=6.49e-05  P=0.00822  P=0.155  P=0.713023  P=0.704  P=1.07e-05  P=0.00142  P=0.493  P=0.00014  P=5.5e-07  P=0.306  P=0.4292  P=1.06e-05  P=0.03289  P=0.7296  P=0.00124  P=2.17e-05 | F (1, 16) =45.03  F (1, 16) =32.08  F (1, 15) =3.999  F (1, 15) =0.004  F (1, 16) =0.018  F (1, 16) =1.598  F (1, 16) =7.572  F (1, 16) =0.065  F (1, 16) =64.160  F (1, 16) =0.335  F (1, 15) =2.996  F (1, 14) =2.451  F (1, 16) =327.98  F (1, 16) =52.323  F (1, 15) =29.602  F (1, 16) =14.64  F (1, 16) =13.77 | P=5.01e-06  P=3.52e-05  P=0.064  P=0.951708  P=0.896  P=0.224283  P=0.01418  P=0.802  P=5.46e-07  P=0.571  P=0.104  P=0.1398  P=4.40e-12  P=2e-06  P=6.82e-05  P=0.00149  P=0.0019 | F (1, 16) =34.24  F (1, 16) =0.41  F (1, 15) =0.057  F (1, 15) =19.154  F (1, 16) =1.309  F (1, 16) =26.070  F (1, 16) =6.172  F (1, 16) =1.534  F (1, 16) =0.713  F (1, 16) =1.790  F (1, 15) =47.648  F (1, 14) =4.720  F (1, 16) =313.89  F (1, 16) =15.600  F (1, 15) =7.481  F (1, 16) =12.27  F (1, 16) =42.24 | P=2.45e-05  P=0.53120  P=0.815  P=0.000542  P=0.269  P=0.000106  P=0.02443  P=0.233  P=0.41098  P=0.200  P=5.04e-06  P=0.0475  P=6.15e-12  P=0.00115  P=0.0153  P=0.00294  P=7.33e-06 |


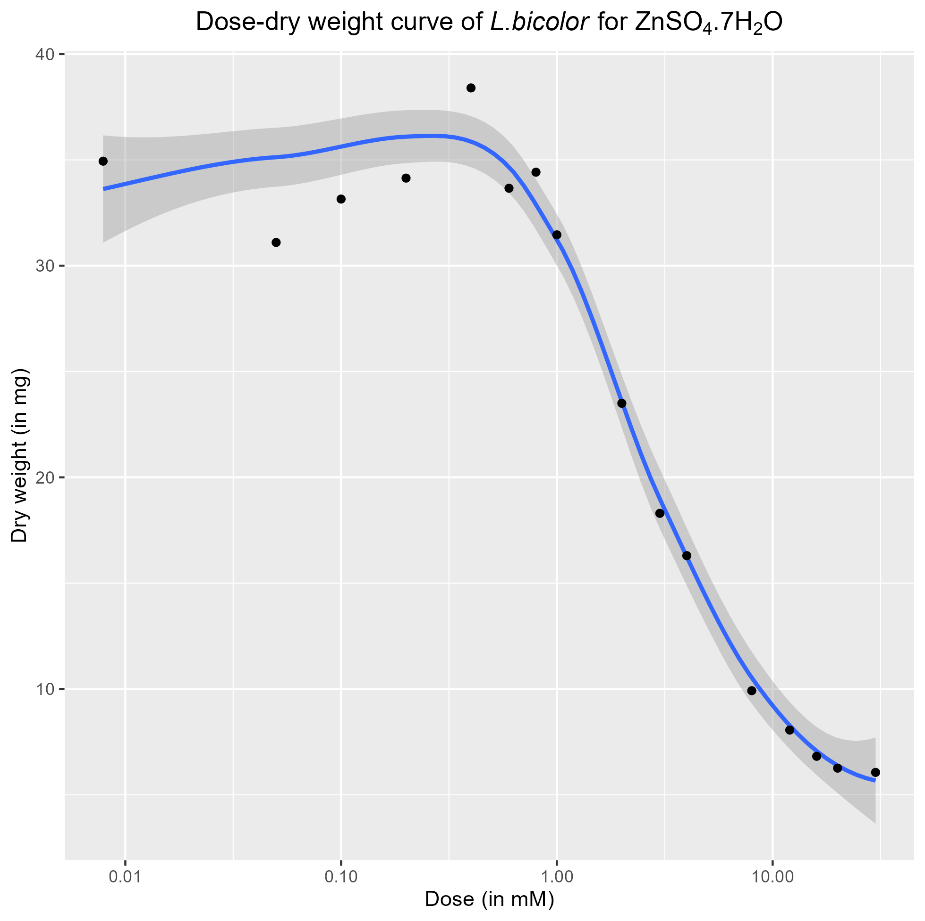


**Supplementary figure 1:** Dose-Response hormetic curve of L. bicolor S238N for ZnSO_4_.7H_2_O. Free-living mycelium was grown on P5 medium containing a range of 16 Zn concentrations between 0.008 mM (control) and 30 mM (n = 5). After three weeks of incubation, mycelium was harvested and lyophilized. The Zn EC50 value was calculated using the dry weight and the drc package in R.

Pellegrin, C., Daguerre, Y., Ruytinx, J., Guinet, F., Kemppainen, M., Frey, N.F.D., et al. (2019). Laccaria bicolor MiSSP8 is a small-secreted protein decisive for the establishment of the ectomycorrhizal symbiosis. *Environ Microbiol* 21(10)**,** 3765-3779. doi: 10.1111/1462-2920.14727.

Zhang, F., Labourel, A., Haon, M., Kemppainen, M., Da Silva Machado, E., Brouilly, N., et al. (2021). The ectomycorrhizal basidiomycete Laccaria bicolor releases a GH28 polygalacturonase that plays a key role in symbiosis establishment. *New Phytol* 233(6)**,** 2534-2547. doi: 10.1111/nph.17940.
